# Poor Sleep Quality Associates With Decreased Functional and Structural Brain Connectivity in Normative Aging: A MRI Multimodal Approach

**DOI:** 10.3389/fnagi.2018.00375

**Published:** 2018-11-20

**Authors:** Liliana Amorim, Ricardo Magalhães, Ana Coelho, Pedro Silva Moreira, Carlos Portugal-Nunes, Teresa Costa Castanho, Paulo Marques, Nuno Sousa, Nadine Correia Santos

**Affiliations:** ^1^Life and Health Sciences Research Institute, School of Medicine, University of Minho, Braga, Portugal; ^2^ICVS/3B's, PT Government Associate Laboratory, Braga, Portugal; ^3^Clinical Academic Center–Braga, Braga, Portugal

**Keywords:** Pittsburgh Sleep Quality Index, PSQI, MRI, whole-brain modeling, brain connectivity, resting-state

## Abstract

Sleep is a ubiquitous phenomenon, essential to the organism homeostasis. Notwithstanding, there has been an increasing concern with its disruption, not only within the context of pathological conditions, such as neurologic and psychiatric diseases, but also in health. In fact, sleep complaints are becoming particularly common, especially in middle-aged and older adults, which may suggest an underlying susceptibility to sleep quality loss and/or its consequences. Thus, a whole-brain modeling approach to study the shifts in the system can cast broader light on sleep quality mechanisms and its associated morbidities. Following this line, we sought to determine the association between the standard self-reported measure of sleep quality, the Pittsburgh Sleep Quality Index (PSQI) and brain correlates in a normative aging cohort. To this purpose, 86 participants (age range 52–87 years) provided information regarding sociodemographic parameters, subjective sleep quality and associated psychological variables. A multimodal magnetic resonance imaging (MRI) approach was used, with whole-brain functional and structural connectomes being derived from resting-state functional connectivity (FC) and probabilistic white matter tractography (structural connectivity, SC). Brain regional volumes and white matter properties associations were also explored. Results show that poor sleep quality was associated with a decrease in FC and SC of distinct networks, overlapping in right superior temporal pole, left middle temporal and left inferior occipital regions. Age displayed important associations with volumetric changes in the cerebellum cortex and white matter, thalamus, hippocampus, right putamen, left supramarginal and left lingual regions. Overall, results suggest that not only the PSQI global score may act as a proxy of changes in FC/SC in middle-aged and older individuals, but also that the age-related regional volumetric changes may be associated to an adjustment of brain connectivity. These findings may also represent a step further in the comprehension of the role of sleep disturbance in disease, since the networks found share regions that have been shown to be affected in pathologies, such as depression and Alzheimer's disease.

## Introduction

Sleep is a recurring and reversible neurobehavioral state that involves reduced responsiveness to external stimuli and is frequently accompanied by postural recumbence and behavioral quiescence (Carskadon and Dement, 2011). Propensity to sleep is determined by the interaction of the circadian rhythm (“process C”), controlled by the suprachiasmatic nucleus, and a sleep homeostatic process (“process S”), that increasingly drives the need for sleep as a function of the time spent awake (Borbély, 1982; Borbély et al., 2016). For the individual to thrive and efficiently cope with the waking day demands, guidelines advise a human adult to sleep 7–9 h every day (Hirshkowitz et al., 2015) and to have sleep continuity parameters (i.e., sleep latency, awakenings >5 min, wake after sleep onset and sleep efficiency) within a specific range (see Ohayon et al., 2017 for details). In fact, having a “good sleep” ensures metabolic homeostasis, cerebral clearance, adequate immune function and overall good cognitive and mental status (Stickgold, 2006; Tononi and Cirelli, 2006; Cirelli and Tononi, 2008; Xie et al., 2013; Irwin, 2015; Freeman et al., 2017; Shokri-Kojori et al., 2018). However, factors, such as inappropriate exposure to light or food, lifestyle schedules, work, or psychological morbidity can interfere with the appropriate timing and duration of the sleep/wake cycle, leading to wide-range adverse effects on health (Schwartz et al., 1999; Stickgold, 2006; Wulff et al., 2010; Archer and Oster, 2015). More so, age also emerges as a critical modifier of sleep-wake patterns, being responsible for a shorter overall sleep duration and increase in sleep fragmentation and fragility, mostly after the fifth decade of age (Mander et al., 2017). In addition, in an increasingly older population (United Nations, Department of Economic and Social Affairs, Population Division, 2013), sleep dissatisfaction is one of the most common complaints in primary care (Aikens and Rouse, 2005) contributing not only for a growth vulnerability to disease, but also for a considerable economic burden, consequence of the costs of sleep aids and work absenteeism (Hillman et al., 2006). In addition, the dyad sleep-depression is of relevant weight since not only sleep problems may underlie an increased risk for the middle-aged or older individuals to be depressed (Almeida and Pfaff, 2005), but are also a robust predictor of incident depression (Livingston et al., 1993; Mallon et al., 2000).

In view of these associations and comorbidities, and because many neurological and psychiatric disorders share underlying brain network disturbances (Buckholtz and Meyer-Lindenberg, 2012; Uhlhaas and Singer, 2012; Deco and Kringelbach, 2014), it is vital to determine how one's sleep complaints and perceptions affect neural circuitries and overall biological systems. On this, the Pittsburgh Sleep Quality Index (PSQI) (Buysse et al., 1989; Landry et al., 2015), a standard subjective measure of overall sleep quality, may provide important and clinically relevant insights. In fact, magnetic resonance imaging (MRI) studies using this composite measure have revealed an association between poor subjective sleep quality and reduced volume within the right superior frontal cortex in cross-sectional analyses, as well as a widespread increased rate of atrophy in frontal, temporal and parietal regions in longitudinal analyses (Sexton et al., 2014). Poor sleep has also been associated with alterations in functional connectivity (FC) of resting state (e.g., default mode network) and attentional networks (Sämann et al., 2010; De Havas et al., 2012; Kaufmann et al., 2016; Scullin, 2017), amygdalar circuits (Shao et al., 2014), as well as in the dorsal nexus-dorsolateral prefrontal cortex connection (Bosch et al., 2013). However, different measures of sleep quality are considered in different studies and most of them tend to evaluate how specific networks behave under strict experimental protocols (e.g., sleep deprivation protocols) or in target populations (e.g., mostly young adults or individuals with sleep pathologies), thus providing a limited view of the occurring mechanisms and processes.

A whole-brain multimodal MRI approach can, therefore, provide new insights into general principles of brain function from health to disease (Deco and Kringelbach, 2014). Hence, in the present study, we used this approach to explore the association between subjective sleep quality (PSQI global score) and FC and structural connectivity (SC). PSQI global score will provide an overall value of the previous month sleep quality. Specifically, we hypothesized that this measure will provide an important overall view of sleep disruption parameters that may affect FC of a network with nodes in frontal, temporal, parietal and occipital regions during restful wake, given the described behavior of these regions during sleep and in sleep pathologies. Furthermore, because PSQI evaluates sleep quality over a 1-month period, we also hypothesized that it will be possible to observe the impact of poor sleep quality in SC.

## Methods

### Ethics statement

The present study was conducted in accordance with the principles expressed in the Declaration of Helsinki (59th amendment) and was approved by the local and national ethics committees. All participants gave informed written consent after the study aims were explained.

### Participants

The participants included in the present study are part of a cohort recruited for the SWITCHBOX Consortium project (www.switchbox-online.eu/). They were randomly selected from Guimarães and Vizela local area health authority registries as representative of the general middle-aged and older Portuguese population for age, gender and education (Costa et al., 2013; Santos et al., 2014). Primary exclusion criteria included inability to understand the informed consent, participant choice to withdraw from the study, incapacity and/or inability to attend the MRI session, dementia and/or diagnosed neuropsychiatric and/or neurodegenerative disorder (medical records). A sample of 120 individuals was invited to the follow-up assessment based on neuropsychological scores. Eighty-six individuals agreed to be re-evaluated (*n* = 17 declined to participate; *n* = 2 could not be reached; *n* = 6 could not be reassessed due to multiple schedules incompatibilities) and from these, 77 were able to perform an MRI scanning session. All MRI acquisitions were obtained during the afternoon to avoid morning and night circadian fluctuations.

### Questionnaires

All participants were asked, in a structured interview format given participants' age and educational status, about the following information: sociodemographic; subjective sleep quality (Pittsburgh Sleep Quality Index, PSQI) (Buysse et al., 1989; Del Rio João et al., 2017); depressive symptoms (Geriatric Depression Scale, GDS) (Yesavage et al., 1982); sleepiness (Epworth Sleepiness Scale, ESS) (Johns, 1991; Santos, 2001). In the questionnaires used, the higher the score, the poorer the outcome. Furthermore, in the present study, PSQI global score was considered as a continuum.

### Actigraphy

Actigraphy is a valuable tool in the study of sleep and circadian rhythms given its ability to measure 24 over 24-h activity (Ancoli-Israel et al., 2003; Landry et al., 2015). ActiSleep+ (Firmware 2.2.1; ActiGraph, LLC, Pensacola, Florida, USA) is a small (4.6 × 3.3 × 1.5 cm), electronic, light weight (19 grams), water proof, tri-axial wrist-worn device, that measures activity “counts” and was initialized at a sample rate of 30 Hz to record activities for free-living conditions. The obtained information was then downloaded using ActiLife 6 software (v 6.9.0; ActiGraph, LLC, Pensacola, FL, USA) and integrated into 60-s epochs for posterior analysis using Cole-Kripke algorithm (Cole et al., 1992). In the present study, 64 participants (of the 86 invited for the study) agreed to use the monitor in the non-dominant wrist for a 7-days period. For each participant, the mean of the 7 days for the following parameters were obtained: total time in bed, total sleep time, sleep latency, sleep efficiency, wake after sleep onset, number of awakenings and time of each awakening. Participants were instructed to never remove the monitor. However, in case of removal, they were instructed to register it in the sleep diary they had to fill for each of the 7 days. Similarities between the period of assessment using ActiSleep+ and the month assessed by PSQI was addressed by asking the participants for that information. For the analysis of the actigraphy information, data from 31 participants was admissible. Exclusion criteria included differences between the actigraphic week and the month assessed by PSQI (*n* = 8); < 6 days of actigraphic data (*n* = 3); sleep diaries not matching quality standards (e.g., not properly filled) (*n* = 22).

### MRI data acquisition

The imaging session was performed at the Hospital of Braga (Braga, Portugal) on a clinical approved Siemens Magnetom Avanto 1.5 T MRI scanner (Siemens Medical Solutions, Erlangen, Germany), using a 12-channel receive-only head-coil. All acquisitions were performed between 2 and 6 p.m. to control for circadian fluctuations. The imaging protocol consisted of three types of acquisitions: structural, resting-state (rs) and diffusion weighted imaging (DWI). For the structural acquisition, a 3D T1-weighted magnetization prepared rapid gradient echo (MPRAGE) sequence was used. The used parameters were: 176 sagittal slices, TR/TE = 2,730/3.48 ms, flip angle = 7°, slice thickness = 1.0 mm, in-plane resolution = 1.0 × 1.0 mm^2^, FoV = 256 × 256 mm. For the rs-fMRI acquisition, a blood oxygen level dependent (BOLD) sensitive echo-planar imaging (EPI) sequence was used and the following parameters were used: 30 axial slices, TR = 2.0 s, TE = 30 ms, flip angle = 90°, slice thickness = 3.5 mm, slice gap = 0.48 mm, in-plane resolution = 3.5 × 3.5 mm^2^, FoV = 224 × 224 mm and 190 volumes. During this resting state scan, participants were instructed to be awake, with their eyes closed, as motionless as possible and they should try not to think of anything in particular and let the mind wander. We choose this approach because our population are middle-aged and older individuals that usually tend to feel very uncomfortable inside the MRI machine, thus moving a lot. Therefore, to reduce the discomfort and movement inside the MRI machine, we choose to ask the participants to stay awake with their eyes closed. In the end of this session, all participants confirmed that they had not fallen asleep. For the Diffusion Weighted Imaging (DWI) scan, a spin-echo echo-planar imaging (SE-EPI) sequence with the following parameters was used: TR = 8,800 ms, TE = 99 ms, FoV = 240 × 240 mm, acquisition matrix = 120 × 120, 61 slices, slice thickness = 2 mm, 30 non-collinear gradient directions with b = 1,000 s/mm^2^, one b = 0 s/mm^2^ acquisition and two as the total number of repetitions.

### MRI data pre-processing

A certified neuro-radiologist visually inspected all acquisitions to confirm that they were not affected by critical head motion (certifying its quality) and that participants had no brain lesions/pathology.

The structural scans of each subject were segmented using FreeSurfer toolkit version 5.1 (http://surfer.nmr.mgh.harvard.edu), which implements a semi-automated segmentation workflow and allows the segmentation of Gray Matter (GM), White Matter (WM) and subcortical regions. For a complete description of the stages of processing implemented in this pipeline see Desikan, Destrieux, and Fischl works (Fischl et al., 2002, 2004; Desikan et al., 2006; Destrieux et al., 2010). Fischl and colleagues (Fischl et al., 2002) validated the procedures against manual segmentations, with robust results across sessions, scanner platforms, updates, and scanner field strengths (Jovicich et al., 2009). The quality of the segmentations was visually inspected and corrections conducted as indicated in Freesurfer guidelines. For the present study, values of intracranial volume (ICV), total gray matter volume (GMV), white matter hypointensities volume (WMSA) and regional brain volumes according to Desikan (Desikan et al., 2006) and Destrieux (Destrieux et al., 2010) segmentations were considered.

rs-fMRI data pre-processing was performed using FMRIB Software Library (FSL v5.07; http://fsl.fmrib.ox.ac.uk/fsl/; Smith et al., 2004) tools. The first five volumes of the rs-fMRI acquisition were removed to allow the habituation of the subjects to the EPI sequence noise and environment, as well as to avoid the spin history effects on the first volumes and the confounding effects over those (Friston et al., 1996). Furthermore, given that our population are middle-aged and older individuals, and likely extra sensitive to the MRI environment, we considered this step to be important. The remaining data was corrected for slice timing (for Siemens interleaved acquisition) followed by head motion correction using the mean image as reference. In order to reduce potential contamination of motion on functional connectivity, motion scrubbing (Power et al., 2012) was also performed, in order to identify and further exclude time-points where head motion could be critical. One subject was excluded for having more than 10 motion-contaminated time-points. Each subject functional dataset was then spatially normalized to the Montreal Neurological Institute (MNI) standard space through an indirect procedure that included: (i) skull stripping of the mean image of the functional acquisition and of the structural acquisition allowing the isolation of brain signal; (ii) rigid-body registration of the mean functional image to the skull stripped structural scan; (iii) affine registration of the structural scan to the MNI T1 template; (iv) non-linear registration of the structural scan to the MNI T1 template using the affine transformation previously estimated as the initial alignment; (v) non-linear transformation of the functional acquisition to MNI standard space trough the concatenation and application of the rigid-body transformation and the non-linear warp followed by resampling to 2 mm isotropic voxel size. Linear regression of motion parameters, mean WM and cerebrospinal fluid (CSF) (extracted using the FSL white matter and CSF tissue priors) signal and motion outliers was performed and the residuals of the regression were band-pass temporal filtered (0.01–0.08 Hz) and used for the subsequent analysis.

Pre-processing of diffusion data was performed using the FSL toolbox FDT (https://fsl.fmrib.ox.ac.uk/fsl/fslwiki/FDT; Behrens et al., 2003). The pre-processing consisted of: (i) eddy current and movement correction and matching rotation of the diffusion directions; (ii) isolation of brain signal by extraction of the skull; for the probabilistic tractography analysis this was followed by (iii) calculation of the non-linear normalization from MNI space to the subjects native space by concatenating an affine registration from structural to the diffusion space to the inverse of the previously described warp map from native structural to MNI space; (iv) the normalization was applied to the AAL atlas to bring all its ROIs to native diffusion space; (v) local modeling of the diffusion parameters was done using bedpostx which runs Markov Chain Monte Carlo sampling to build voxel wise distributions of the diffusion parameters; for the voxel wise diffusion parameters analysis (ii) was followed by (vi) the diffusion tensor was fitted to the data and the scalar maps were computed. This was achieved using DTIFIT that is part of the FDT Toolbox. DTIFIT fits a diffusion tensor model at each voxel and generates the scalar maps of fractional anisotropy (FA) and mean diffusivity (MD), and eigenvector and eigenvalue maps. Axial Diffusivity (AD) scalar map was defined as the principal diffusion eigenvalue (L1) and radial diffusivity (RD) as the mean of the second and third eigenvalues ((L2 + L3)/2).

### MRI data analysis

A connectomics approach was used for resting state functional and diffusion structural connectivity analysis and the networks were built using the Anatomical Automatic Labeling (AAL) atlas. For the functional data analysis, the mean signal across time was extracted for each of the 116 ROIs and for each subject. Then, Pearson correlations between each possible pair of regions were computed and a symmetric adjacency matrix R, where each entry rij represents the Pearson correlation coefficient between the time series of region i and j, was obtained. These matrices were transformed into Z-score matrices by the application of Fisher's r-to-Z transformation to assure the normality of the correlation coefficients. To increase the statistical power of the analysis, the network-based statistic (NBS) procedure implemented in the NBS toolbox (https://sites.google.com/site/bctnet/comparison/nbs) was used (Zalesky et al., 2010). NBS evaluates the null hypothesis at the level of interconnected edges (i.e., subnetworks) surviving a predefined primary threshold (instead of considering the null hypothesis at the single edge level). The null hypothesis assumes that a sub-network with similar number of edges, surpassing the primary threshold, occurs by chance. It is recommended the use of different primary thresholds in order to capture different effects. In the present study, three different primary thresholds were used (*P* < 0.01, *P* < 0.005, *P* < 0.001) to capture less pronounced but more extent effects (less stringent primary threshold—*P* < 0.01) as well as localized and pronounced effects (most stringent threshold—*P* < 0.001). Five thousand permutations were performed and networks were considered significant at a network size corrected level of *P* < 0.05. To simplify visualization of the results, when similar networks were found across the threshold levels, we favor the presentation of the network surviving the higher threshold. BrainNet viewer (http://www.nitrc.org/projects/bnv/) was used for visualization purposes.

For the diffusion data probtrackx from the FDT toolbox (Behrens et al., 2007) was used to estimate the structural connectivity by calculating the number of streamlines connecting each pair of ROIs from the atlas, through sampling the principle directions previously calculated at each voxel. A total of 5,000 streamlines were attempted per-voxel. This resulted in a matrix representing the number of streamlines reaching from one ROI to the other. This matrix was normalized by first dividing each line by the number of voxels × number of streamlines and then the upper and lower triangles were averaged to give an undirected connectivity. To filter the connectivity matrix, keeping only connections significantly different from zero, a one-sample *t*-test was done at each connection. Only connections with a *p* lower than 0.01 were considered. To test the structural networks the same procedures for NBS described for functional connectivity were applied. Voxel-wise statistical analysis of scalar maps was performed using tract-based spatial statistics (TBSS) procedures (Smith et al., 2006), also implemented in FSL, following thresholding option TFCE (Threshold-Free Cluster Enhancement). For TBSS, first, the FA maps of each participant were slightly eroded and the end slices were zeroed, so that potential outliers from diffusion tensor fitting could be removed. Then, a non-linear registration was applied to align all FA images to a 1 × 1 × 1 mm standard space. In order to perform this, the FA image from each subject was non-linearly registered to each other in order to find the most representative one (i.e., the one that requires the least warping to align all images) that served as the study specific template. This template image was then affine transformed into Montreal Neurological Institute (MNI) 152 standard space and each FA map was transformed into standard space by combining the non-linear transformation to the FA target with the affine transformation into MNI space. Then, all FA images were averaged and the resulting image was skeletonized. The resultant skeleton image was thresholded at 0.3 so that skeleton regions including multiple tissue types could be removed. Finally, all scalar maps (FA, MD, AD, and RD) were projected onto the mean FA skeleton using the transformations applied to the FA images.

### Statistical analysis

Statistical analysis using SPSS version 22 (IBM, SPSS, Chicago, IL, USA) was used to determine the association between psychological, actigraphic, clinical and sociodemographic variables. The normality assumption for each variable was tested and non-parametric tests used when the assumption was not met. Bonferroni correction was used for multiple comparison testing and the significance level was set at *p* < 0.003.

For the statistical testing of FC and SC it were considered: a Network Based Statistics (NBS, https://sites.google.com/site/bctnet/comparison/nbs; Zalesky et al., 2010) approach, corrected for the size of the network, and a model that included GDS total score, PSQI global score, the interactions “PSQI × GDS,” “PSQI × Age,” and “PSQI × Sex” (as independent variables), age, sex, and years of education (as covariates). NBS tests the hypothesis in two stages: first, at each possible individual network connection by applying a user defined significance threshold; second, by identifying sub-networks composed of connections whose significance surpasses the threshold and determining its significance according to the network size. The sub-networks significance was calculated by comparing their sizes to the distribution of the size of sub-networks obtained through 5,000 random permutations of the original hypothesis. Because different thresholds can yield topologically different networks, three thresholds were tested at *p* < 0.01, *p* < 0.005, and *p* < 0.001. If the networks found at different thresholds are found to be equivalent, only the most significant one will be presented and discussed. The statistical analysis of the skeletonized maps of FA, MD, AD and RD was performed using permutation-based cross-subject statistics implemented in randomize, distributed with FSL. The model created was then used to test the main effect of PSQI variable and the PSQI by age, sex, years of formal education and GDS interactions. Ten thousand permutations were performed in the inference of each contrast of interest.

Volumetric data statistical analysis was performed ROI-wise using Matlab R2009b software (www.mathworks.com). A mix of Desikan (Desikan et al., 2006) and Destrieux (Destrieux et al., 2010) areas were considered for the subdivision of brain regions to be analyzed and a regression model considering sex, age, education, GDS total score, PSQI global score, ICV, the interactions “PSQI × Age” and “PSQI × sex” (independent variables) and the ROI volume of each area (dependent variable) was used. The choice of this regional brain areas was performed considering the most relevant areas regarding sleep that have been considered in different studies. White matter hypointensities and non-white matter hypointensities were also analyzed under this model. Bonferroni correction was used for multiple comparisons testing.

## Results

### Cohort characterization

The study sample was composed by 46 males and 40 females, with a mean age of 67.40 (±8.155) years and a median of 4 (IQR = 3) years of education. More than half of the participants were married (77.9%), retired (75.6%) and presented a body mass index (BMI) above the considered normal range (93% with BMI above 25). 20% of the participants were on benzodiazepines use. See Table 1 for more details.

**Table 1:** Cohort characterization in terms of socio-demographic factors and clinical and psychological parameters.

		***n* (%)**	**Mean (±SD)**
**SOCIO-DEMOGRAPHIC PARAMETERS**			
Age			67.4 (±8.155)
Sex	Male	46 (53.5%)
	Female	40 (46.5%)
Education			4 (3)^#^
Marital status	Single	1 (1.2%)
	Married	67 (77.9%)
	Divorced	3 (3.5%)
	Widowed	15 (17.4%)
Household	Alone	9 (10.5%)
	Partner	38 (44.2%)
	Family	39 (45.3%)
Occupational status	Retired	65 (75.6%)
	Employed	15 (17.4%)
	Unemployed	6 (7.0%)
**CLINICAL PARAMETERS**			
Weight (kg)			74.15 (±11.175)
Height (cm)			1.581 (±0.091)
BMI			29.40 (±3.539)
BP Systolic			133.50 (±39.827)
BP Diastolic			71.34 (±31.610)
**Medication**^¥^
Insulin	Yes	1 (1.2%)
Anti-diabetic	Yes	16 (18.6%)
Anti-hypertensive	Yes	46 (53.5%)
Aspirin	Yes	13 (15.1%)
Anti-Inflamatory non-histeroid	Yes	11 (12.8%)
Colestherol	Yes	39 (45.3%)
Psychopharmacological	Yes	21 (24.4%)
Benzodiazepines_CNS suppressors	Yes	20 (23.3%)
Proton Pump Inibitors	Yes	28 (32.6%)
Anti-Acids_H2 inibitors	Yes	4 (4.7%)
**PSYCHOLOGICAL PARAMETERS**			
PSQI			7 (7.25)^#^
GDS			8 (10)^#^
ESS			7 (7.75)^#^

¥ 5.8% of participants do not present information regarding medication intake;

# *Median (IQR)*.

*BP Systolic, systolic blood pressure; BP Diastolic, diastolic blood pressure; CNS suppressors, central nervous system suppressors; PSQI, Pittsburgh Sleep Quality Index global score; GDS, Geriatric Depression Scale; ESS, Epworth Sleepiness Scale*.

### Subjective sleep quality associates with actigraphic parameters

Almost half (49%) of the participants had poor sleep quality (PSQI global score higher than 5). No statistically significant differences were found for PSQI global score and sex (*U* = 766.00, *p* = 0.180). No statistically significant association was found between PSQI global score and depressive symptomatology (GDS; rho = 0.279, *p* = 0.011) but depressive symptoms and PSQI subdomains “Day Dysfunction” (rho = 0.342, *p* = 0.002), “Sleep Disturbance” (rho = 0.377, *p* < 0.001), and “Medication” (rho = 0.421, *p* < 0.001) were found to be associated. Furthermore, education was negatively correlated with the frequency of sleep medication intake (rho = −0.337, *p* = 0.002) and with depressive symptoms (rho = −0.474, *p* < 0.001). Results are presented in detail in Table 2.

**Table 2:** Correlation between subjective sleep quality and demographic, clinical, and psychological parameters.

	**Age**	**Education**	**Weight**	**BMI**	**BP Systolic**	**BP Diastolic**	**GDS**	**Epworth**	**PSQI_Total_**	**PSQI_Quality_**	**PSQI_Latency_**	**PSQI_Duration_**	**PSQI_Efficiency_**	**PSQI_Disturbance_**	**PSQI_Medication_**	**PSQI_Day Disfunction_**
Age															
Education	−0.341***														
Weight	−0.209	0.190													
BMI	0.090	−0.213*	0.666***												
BP Systolic	0.227*	−0.059	0.053	0.217*											
BP Diastolic	−0.451***	0.248*	0.278**	0.082	0.321***										
GDS	0.007	−0.474***	0.016	0.350***	0.063	−0.015									
Epworth	−0.085	0.009	0.273*	0.314**	−0.157	−0.006	0.254*								
PSQI_Total_	−0.075	−0.167	0.014	0.127	0.179	0.234*	0.279*	−0.060							
PSQI_Quality_	−0.107	−0.031	0.139	0.190	0.108	0.246*	0.213	0.038	0.690***						
PSQI_Latency_	−0.021	−0.135	−0.070	0.021	0.192	0.223*	0.248*	−0.141	0.788***	0.533***					
PSQI_Duration_	−0.224*	0.069	−0.031	−0.115	−0.088	0.131	−0.051	−0.122	0.604***	0.398***	0.358***				
PSQI_Efficiency_	0.021	−0.111	0.106	0.195	0.050	0.110	0.091	−0.041	0.679***	0.320***	0.531***	0.578***			
PSQI_Disturbance_	−0.133	−0.105	0.140	0.203	0.047	0.140	0.377***	0.322***	0.589***	0.393***	0.398***	0.215*	0.256*		
PSQI_Medication_	0.038	−0.337***	−0.115	0.096	0.149	0.053	0.421***	−0.002	0.595***	0.340***	0.386***	0.127	0.150	0.430***	
PSQI_Day Disfunction_	−0.131	−0.188	0.139	0.322***	0.123	0.105	0.342***	0.048	0.498***	0.291**	0.268*	0.226*	0.215*	0.381***	0.387***

Statistic used: Spearman's rho;

* Correlation is significant at the 0.05 level (2-tailed);

** Correlation is significant at the 0.01 level (2-tailed);

*** *Correlation is significant at the 0.003 level (Bonferroni correction)*.

*BMI, body mass index; BD Systolic, systolic blood pressure; BP Diastolic, diastolic blood pressure; PSQI, Pittsburgh Sleep Quality Index; GDS, Geriatric Depression Scale; ESS, Epworth Sleepiness Scale*.

*Bold used to highlight all values of p within the different statistical significant thresholds*.

To complement subjective sleep quality information, we asked a random sub-sample of participants to wear an actigraphic device concomitantly to the fill of a sleep diary for a 7-days period. No differences were found regarding age, depressive symptomatology and subjective sleep quality between this sub-sample and the total cohort (Table 3). Actigraphy results revealed an association between the actigraphy-derived measures “Total Time in Bed” (TTB) and “Total Sleep Time” (TST) and the PSQI subdomain “Efficiency” (rho_TTB_ = 0.583, *p* = 0.001; rho_TST_ = 0.597, *p* < 0.001). Results also showed that sleepiness and the actigraphy-derived “Wake After Sleep Onset” were correlated (WASO, rho = 0.550, *p* = 0.002; Table 4).

**Table 3:** Differences regarding age, PSQI global score, GDS, and ESS between the original cohort and the subsample that used actigraphy.

	**All cohort**		**Sub-sample**		***P***
	**Mean (±SD)**	**Min–Max**	**Mean (±SD)**	**Min–Max**
Age	67.4 (±8.16)	52–84	65.48 (±8.46)	52–84	0.26
PSQI_totalscore_	7 (7.25)^#^	0–20	7 (10)^#^	0–20	0.1
GDS	8 (10)^#^	1–28	8 (11)^#^	0–28	0.88
EPW	7 (7.75)^#^	0–20	6.5 (7)^#^	0–18	0.92

# *Median (IQR)*.

**Table 4:** Correlation between subjective sleep quality, sleepiness, depressive symptoms, and actigraphic parameters.

	**PSQI_Total_**	**PSQI_Quality_**	**PSQI_Latency_**	**PSQI_Duration_**	**PSQI_Efficiency_**	**PSQI_Disturbance_**	**PSQI_Medication_**	**PSQI_Day Disfunction_**	**Epworth**	**GDS**	**Actigraphy_Bed Time_**	**Actigraphy_Wake Time_**	**Actigraphy_Latency_**	**Actigraphy_Efficiency_**	**Actigraphy_TTB_**	**Actigraphy_TST_**	**Actigraphy_WASO_**	**Actigraphy_#Awkenings_**
PSQI_total_																		
PSQI_Quality_	0.811***
PSQI_Latency_	0.827***	0.712**
PSQI_Duration_	0.624***	0.499**	0.401*
PSQI_Efficiency_	0.686***	0.573**	0.700**	0.513**
PSQI_Disturbance_	0.582***	0.485**	0.549**	0.217	0.277
PSQI_Medication_	0.550***	0.388*	0.264	0.275	0.393*	0.297
PSQI_Day Disfunction_	0.580***	0.294	0.294	0.292	0.117	0.473**	0.529**
Epworth	−0.065	−0.051	−0.090	−0.118	−0.034	0.278	0.063	0.248
GDS	0.477***	0.391*	0.356*	0.201	0.480**	0.267	0.675***	0.357*	0.142
Actigraphy_BedTime_	−0.117	0.126	−0.272	0.160	−0.281	−0.211	−0.175	−0.126	−0.046	−0.408*
Actigraphy_WakeTime_	0.238	0.321	0.276	−0.060	0.387*	0.115	0.094	0.105	0.282	0.127	0.097
Actigraphy_Latency_	−0.075	−0.095	−0.110	−0.166	−0.039	0.064	0.025	0.013	0.232	0.188	−0.249	0.190
Actigraphy_Efficiency_	0.123	0.105	0.117	0.098	0.043	0.125	0.175	0.035	−0.483**	0.064	0.004	−0.252	−0.589**
Actigraphy_TTB_	0.382*	0.202	0.495**	−0.129	0.583***	0.289	0.324	0.202	0.220	0.466**	−0.688***	0.574***	0.307	−0.204
Actigraphy_TST_	0.432*	0.241	0.498**	−0.070	0.597***	0.347	0.456**	0.277	0.150	0.589***	−0.688***	0.462**	0.081	0.142	0.912***
Actigraphy_WASO_	0.105	0.065	0.142	−0.063	0.275	0.053	0.003	0.092	0.550***	0.152	−0.219	0.535***	0.547**	−0.886***	0.572***	0.269
Actigraphy_#Awkenings_	0.230	0.236	0.188	0.199	0.319	0.077	0.082	0.100	0.277	0.122	−0.161	0.395*	0.508**	−0.638***	0.455*	0.194	0.721***
Actigraphy_AvgTAwkenings_	−0.068	−0.177	0.034	−0.243	0.055	−0.065	−0.068	−0.064	0.352	0.096	−0.312	0.097	0.029	−0.458**	0.323	0.236	0.500**	−0.133

Statistic used: Spearman's rho;

* Correlation is significant at the 0.05 level (2-tailed);

** Correlation is significant at the 0.01 level (2-tailed);

*** Correlation is significant at the 0.003 level (Bonferroni correction)

*PSQI, Pittsburgh Sleep Quality Index; Epworth, Epworth Sleepiness Scale; GDS, Geriatric Depression Scale*.

*Actigraphy: Bed Time, time participant went to bed; Wake Time, time participant got up; TTB (Total Time in Bed), total amount of time spent in bed; TST (Total Sleep Time), number of minutes scored as sleep during time-in-bed; Latency, number of minutes from lights out until the first epoch of recorded sleep; Efficiency, calculated as (TST/TTB) × 100; WASO (Wake After Sleep Onset), number of minutes scored as wake after sleep onset; #Awakenings, total number of transitions to wake from sleep; Avg T Awakenings, average time of each transitions to wake from sleep*.

*Bold used to highlight all values of p within the different statistical significant thresholds*.

### Brain connectivity is associated with subjective sleep quality

**Figure 1:**
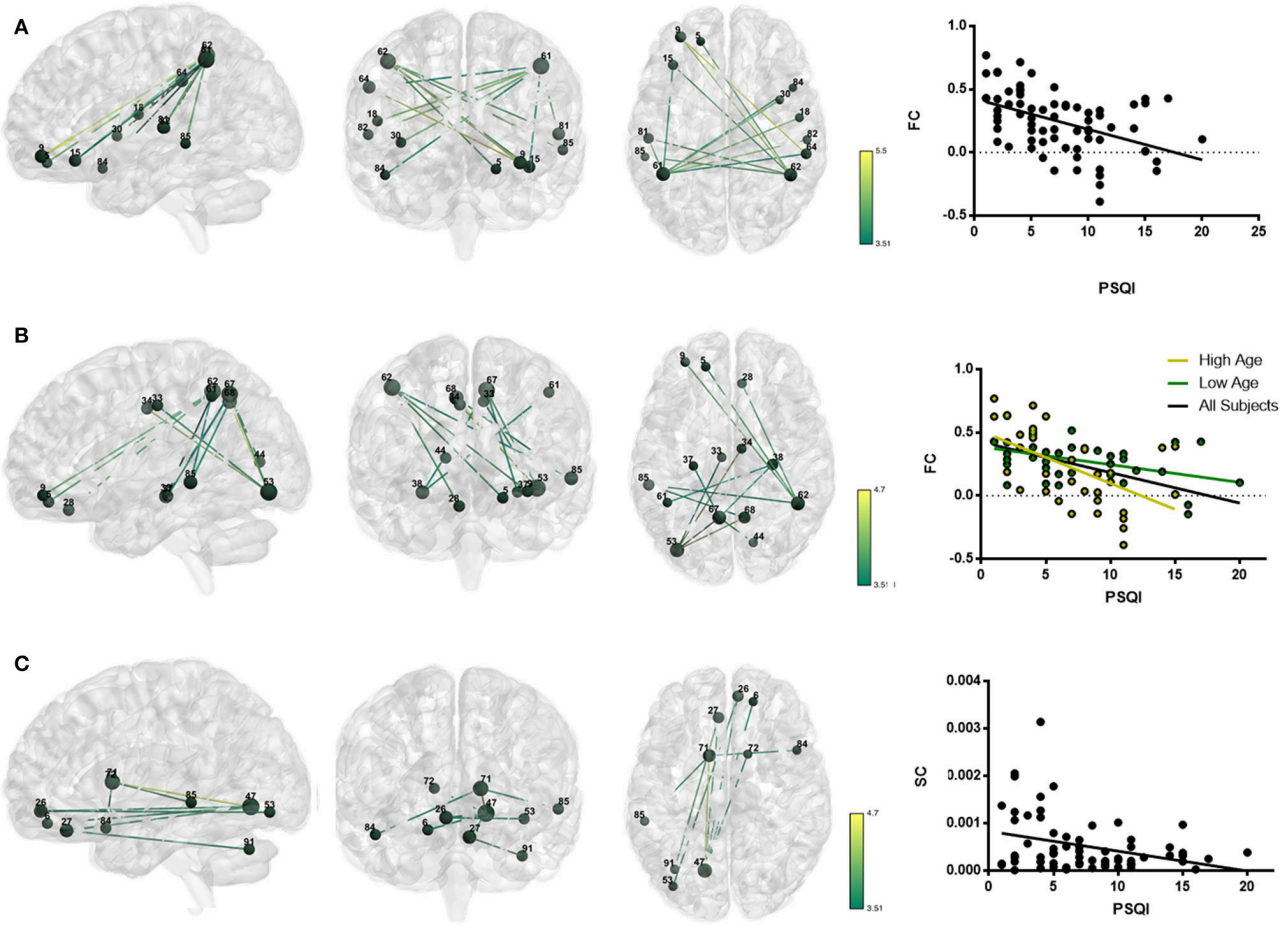
Whole-brain networks with altered functional and structural connectivity (FC and SC) in association with subjective sleep quality using *p* = 0.001 as primary threshold. **Color scheme:** higher connectivity from green to yellow; Nodes: Larger nodes, higher structural connectivity. **(A)** FC in association with PSQI (controlled for age, sex, and GDS); **(B)** FC in association with “Age × PSQI interaction” (controlled for sex and GDS); **(C)** SC in association with PSQI (controlled for age, sex and GDS). **Numbers' legend:** (5) Frontal_Sup_Orb_L; (6) Frontal_Sup_Orb_R; (9) Frontal_Mid_Orb_L; (15) Frontal_Inf_Orb_L; (18) Rolandic_Oper_R; (26) Frontal_Med_Orb_R; (27) Rectus_L; (28) Rectus_R; (30) Insula_R; (33) Cingulum_Mid_L; (34) Cingulum_Mid_R; (37) Hippocampus_L; (38) Hippocampus_R; (44) Calcarine_R; (47) Lingual_L; (53) Occipital_Inf_L; (61) Parietal_Inf_L; (62) Parietal_Inf_R; (64) SupraMarginal_R; (67) Precuneus_L; (68) Precuneus_R; (71) Caudate_L; (72) Caudate_R; (81) Temporal_Sup_L; (82) Temporal_Sup_R; (84) Temporal_Pole_Sup_R; (85) Temporal_Mid_L; (91) Cerebelum_Crus1_L.

**Figure 2:**
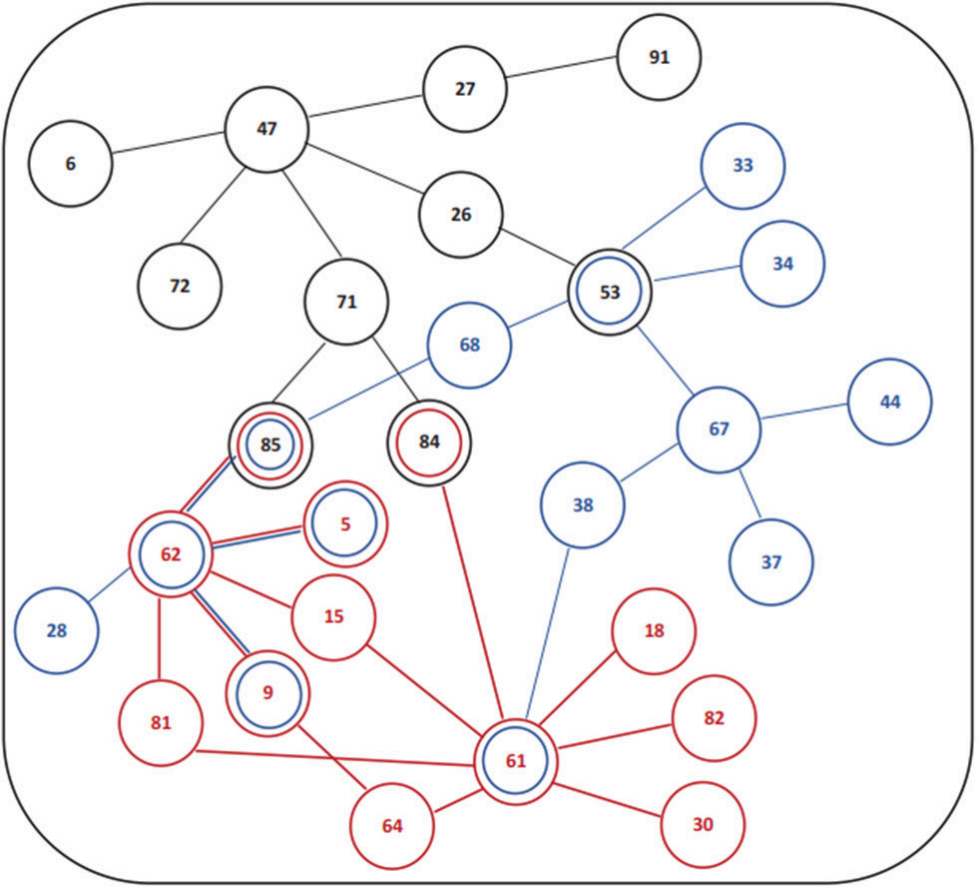
Schematic design or functional and structural connectivity in association with subjective sleep quality. (Red) Association PSQI and FC; (Blue) Association FC and “Age × PSQI” interaction; (Black) Association PSQI and SC. **Number legends**: (5) Frontal_Sup_Orb_L; (6) Frontal_Sup_Orb_R; (9) Frontal_Mid_Orb_L; (15) Frontal_Inf_Orb_L; (18) Rolandic_Oper_R; (26) Frontal_Med_Orb_R; (27) Rectus_L; (28) Rectus_R; (30) Insula_R; (33) Cingulum_Mid_L; (34) Cingulum_Mid_R; (37) Hippocampus_L; (38) Hippocampus_R; (44) Calcarine_R; (47) Lingual_L; (53) Occipital_Inf_L; (61) Parietal_Inf_L; (62) Parietal_Inf_R; (64) SupraMarginal_R; (67) Precuneus_L; (68) Precuneus_R; (71) Caudate_L; (72) Caudate_R; (81) Temporal_Sup_L; (82) Temporal_Sup_R; (84) Temporal_Pole_Sup_R; (85) Temporal_Mid_L; (91) Cerebelum_Crus1_L.

The association of subjective sleep quality with brain connectivity was then assessed using a whole-brain multimodal approach with a restrict connection threshold of *p* < 0.001 and correcting for the size of the network. Results revealed that individuals with poor sleep quality (i.e., higher PSQI global score) had decreased FC in a network with its principal nodes in bilateral inferior parietal regions (nodes 61 and 62) projecting to frontal (nodes 5, 9, and 15), temporal (nodes 81, 82, 84, and 85) and supramarginal (node 64) regions, as well as to the insula (node 30) and Rolandic Operculum (node 18) (network *p* = 0.034, 13 connections; Figure 1A; Table 5). A statistically significant negative association was also found between “Age × PSQI” interaction and FC of a network with its principal nodes in the left inferior occipital (node 53) and left inferior parietal regions (node 61), as well as with the precuneus (nodes 67 and 68) (network *p* = 0.0042, 13 connections; Figure 1B; Table 5). This network also comprised nodes in the right hippocampus (node 38), left middle temporal region (node 85), right inferior parietal region (node 62) and right rectus (node 28). Further analysis of “Age × PSQI” interaction revealed an inflection point at 67 years of age (Figure 1B), after which variations in PSQI global scores produced higher FC alterations when compared to the younger individuals of the cohort. In addition, the association between the amount of fibers connecting different brain areas and the PSQI global score revealed that the most altered connections involved left lingual (node 47), left caudate (node 71), right orbital medial frontal (node 26) and left rectus (node 27) nodes (network *p* = 0.0344; Figure 1C; Table 5). We next explored the structural connection patterns within the patterns of functional interactions. Data showed that there was no overlapping network of the SC and FC associated to subjective sleep quality, but only specific nodes, namely, the right superior temporal pole, left middle temporal and left inferior occipital regions (Figure 2). The left middle temporal region was an overlapping node for the three networks found.

**Table 5:** Functional and structural connectivity results for the tested thresholds.

	**Area**	**FC**						**SC**		
		**PSQI negative**			PSQI*Age negative			**PSQI negative**		
		**0.01 (*p* = 0.046)**	**0.005 (*p* = 0.071)**	**0.001 (*p* = 0.034)**	**0.01 (*p* = 0.006)**	**0.005 (*p* = 0.011)**	**0.001 (*p* = 0.042)**	**0.01 (*p* = 0.049)**	**0.005 (*p* = 0.077)**	**0.001 (*p* = 0.0344)**
5	Frontal_Sup_Orb_L	13.2	7.5649	4.2085	3.7706	3.7706	3.7706		
6	Frontal_Sup_Orb_R							0	3.5448	3.5448
9	Frontal_Mid_Orb_L	15.987	10.334	10.334	36.922	25.928	3.8624		
15	Frontal_Inf_Orb_L	16.827	14.078	7.5094					
18	Rolandic_Oper_R	10.647	10.647	4.2953					
26	Frontal_Med_Orb_R							7.3588	22.983	7.3588
27	Rectus_L							7.4671	7.4671	7.4671
28	Rectus_R				33.515	19.731	3.8328		
30	Insula_R	13.502	13.502	4.3353					
33	Cingulum_Mid_L				21.283	13.053	3.7055		
34	Cingulum_Mid_R				26.715	4.2642	4.2642		
37	Hippocampus_L				21.283	13.053	3.7055		
38	Hippocampus_R				26.715	4.2642	4.2642		
44	Calcarine_R				14.522	6.451	3.5345		
47	Lingual_L							11.965	22.11	18.981
53	Occipital_Inf_L				31.134	25.598	16.426	3.6763	13.409	3.6763
61	Parietal_Inf_L	63.511	52.564	27.808	39.472	19.801	3.4924		
62	Parietal_Inf_R	57.321	49.056	21.498	43.491	24.347	14.958		
64	SupraMarginal_R	38.545	27.308	8.876					
67	Precuneus_L				69.115	36.177	14.232		
68	Precuneus_R				55.128	44.089	8.1211		
71	Caudate_L							8.3909	15.375	12.011
72	Caudate_R							0	9.6374	3.4716
81	Temporal_Sup_L	28.369	17.374	8.2434					
82	Temporal_Sup_R	10.601	7.9123	4.5051					
84	Temporal_Pole_Sup_R	9.0372	3.5454	3.5454				0	6.5682	3.6199
85	Temporal_Mid_L	12.841	7.3347	4.1847	18.335	12.931	6.9458	3.807	3.807	3.807
91	Cerebelum_Crus1_L							3.7688	3.7688	3.7688

*FC, functional connectivity; SC, structural connectivity. The underlined values represent the higher t values for each node*.

### White matter properties and subjective sleep quality

No statistically significant results were found regarding the association of white matter properties and sleep quality.

### Volumetric changes and subjective sleep quality

Age and depressive symptomatology, but not PSQI global score, were significantly correlated with regional brain volumes (Table 6). Specifically, an increase in age was associated to a decrease in the volumes of cerebellum white matter (left: *r*^2^ = 0.742, *p* < 0.001; right: *r*^2^ = 0.674, *p* < 0.001), cerebellum cortex (left: *r*^2^ = 0.643; *p* = 0.01; right: *r*^2^ = 0.632, *p* = 0.013), thalamus (left: *r*^2^ = 0.531; *p* = 0.009; right: *r*^2^ = 0.543; *p* = 0.001), hippocampus (left: *r*^2^ = 0.463; *p* = 0.011; right: *r*^2^ = 0.421; *p* = 0.004), right putamen (*r*^2^ = 0.414; *p* = 0.033), left lingual (*r*^2^ = 0.341; *p* = 0.014) and left supramarginal (*r*^2^ = 0.472; *p* = 0.032). Furthermore, while increases in white matter hypointensities (*r*^2^ = 0.427; *p* = 0.003) were associated to increased age, a negative association is observed between the depressive symptomatology measured by GDS and lower left putamen volumes (*r*^2^ = 0.415; *p* = 0.046).

**Table 6:** Regional brain volumes association with age, sex, subjective sleep quality, and depressive symptoms.

	***r*2**	***β***							***p*****-value**							***p*****-value adjusted**						
		**PSQI**	**Age**	**Sex**	**GDS**	**Education**	PSQI*Age	PSQI*Sex	**PSQI**	**Age**	**Sex**	**GDS**	**Education**	PSQI*Age	PSQI*Sex	**PSQI**	**Age**	**Sex**	**GDS**	**Education**	PSQI*Age	PSQI*Sex
Left-Cerebellum-White-Matter	0.742	−29.51	−148.96	−345.58	−71.84	−27.68	8.61	67.47	0.516	**0.000**	0.363	**0.004**	0.612	**0.043**	0.268	14.792	**0.000**	9.899	0.240	14.065	2.289	11.789
Left-Cerebellum-Cortex	0.643	−30.95	−208.06	−2787.04	−190.36	−85.24	7.81	71.24	0.810	**0.000**	**0.012**	**0.007**	0.582	0.512	0.679	10.752	**0.011**	0.664	0.411	15.005	7.817	12.160
Left-Thalamus-Proper	0.531	47.12	−36.25	−34.31	−20.73	42.12	2.43	−54.05	**0.037**	**0.000**	0.853	0.084	0.118	0.237	0.072	2.109	**0.009**	4.933	3.445	6.464	8.534	3.968
Left-Caudate	0.357	28.52	14.42	74.35	8.29	29.42	2.07	−45.71	**0.041**	**0.013**	0.518	0.259	0.077	0.114	**0**.**014**	2.268	0.516	11.181	7.725	4.541	5.260	0.882
Left-Putamen	0.415	57.47	−24.81	−62.18	−40.38	−0.13	2.56	−43.91	**0.009**	**0.006**	0.728	**0.001**	0.996	0.197	0.128	0.542	0.256	7.948	**0.046**	2.945	8.097	6.664
Brain-Stem	0.676	7.30	−54.59	−1891.21	−72.86	−122.26	13.32	70.85	0.914	**0.050**	**0.001**	**0.046**	0.133	**0.035**	0.431	6.261	1.612	0.078	2.333	7.074	1.949	15.531
Left-Hippocampus	0.463	27.40	−21.41	−245.12	−19.05	14.25	1.61	−20.37	**0.043**	**0.000**	**0.033**	**0.010**	0.396	0.199	0.254	2.324	**0.011**	1.739	0.549	15.446	8.097	11.418
Left-Amygdala	0.364	9.77	−6.24	−159.78	−6.09	3.14	1.04	−12.64	0.156	**0.028**	**0.007**	0.103	0.714	0.106	0.168	7.102	0.989	0.426	3.813	12.484	4.991	8.411
CSF	0.234	9.38	13.10	−0.32	8.70	−0.04	−1.01	−13.43	0.446	**0.012**	0.998	0.180	0.998	0.378	0.414	14.722	0.482	1.980	6.289	1.992	8.244	15.304
Left-Accumbens-area	0.355	2.15	−4.41	−46.63	−3.72	5.24	0.07	2.04	0.555	**0.004**	0.128	0.059	0.233	0.842	0.674	14.438	0.193	6.158	2.838	10.707	6.679	12.811
Left-choroid-plexus	0.381	−0.87	9.30	67.24	1.44	5.75	−0.34	−2.01	0.920	**0.010**	0.350	0.753	0.577	0.664	0.861	4.576	0.415	10.438	5.107	15.540	8.573	7.632
Right-Cerebellum-White-Matter	0.674	−60.02	−144.31	−475.94	−54.93	−39.11	5.46	92.32	0.256	**0.000**	0.281	**0.054**	0.536	0.263	0.192	9.996	**0.000**	10.395	2.647	16.549	8.431	9.232
Right-Cerebellum-Cortex	0.632	−42.01	−211.84	−3111.87	−238.33	−96.27	13.34	92.53	0.753	**0.000**	**0.007**	**0.001**	0.548	0.281	0.604	12.559	**0.013**	0.407	0.081	16.090	8.707	14.883
Right-Thalamus-Proper	0.543	41.27	−40.45	−107.93	−20.36	44.22	3.69	−30.80	0.061	**0.000**	0.551	0.082	0.093	0.069	0.290	3.138	**0.001**	10.245	3.458	5.206	3.370	12.458
Right-Caudate	0.317	34.67	12.11	124.09	0.48	31.06	3.33	−37.78	**0.019**	**0.046**	0.308	0.951	0.077	**0.018**	0.053	1.136	1.512	10.486	2.754	4.541	1.033	2.977
Right-Putamen	0.414	59.76	−30.02	−241.62	−29.33	−13.46	4.74	−35.91	**0.005**	**0.001**	0.161	**0.009**	0.582	**0.017**	0.188	0.282	**0.033**	7.560	0.495	14.545	0.980	9.220
Right-Hippocampus	0.421	28.65	−23.86	−245.99	−14.05	6.00	1.53	−22.60	**0.040**	**0.000**	**0.037**	0.063	0.728	0.237	0.220	2.268	**0.004**	1.907	2.945	11.640	8.534	10.125
Right-Amygdala	0.281	10.98	−4.66	−157.86	0.08	8.67	0.57	−18.83	0.202	0.185	**0.032**	0.986	0.421	0.479	0.103	8.693	4.632	1.739	1.902	15.339	8.163	5.546
Right-Accumbens-area	0.265	4.03	−3.83	−32.21	−1.11	0.10	0.56	−2.14	0.213	**0.005**	0.233	0.517	0.980	0.064	0.618	8.933	0.217	9.545	8.908	4.894	3.193	14.498
Right-choroid-plexus	0.400	−5.46	19.26	116.42	4.87	13.92	−0.96	−0.96	0.610	**0.000**	0.192	0.389	0.278	0.338	0.946	14.041	**0.003**	8.622	8.168	12.514	8.795	2.835
WM-hypointensities	0.427	−65.80	75.55	221.39	38.98	−46.87	−9.25	12.96	0.113	**0.000**	0.508	0.069	0.343	**0.020**	0.809	5.414	**0.003**	11.181	3.171	14.396	1.113	8.377
non-WM-hypointensities	0.112	−1.31	2.02	−0.58	0.93	1.77	−0.13	−1.12	0.407	**0.003**	0.965	0.290	0.351	0.375	0.595	14.655	0.124	3.614	7.593	14.396	8.533	15.437
Optic-Chiasm	0.280	0.58	−0.78	−46.81	−0.77	−3.70	0.23	1.97	0.812	0.421	**0.021**	0.544	0.196	0.299	0.546	10.530	4.530	1.169	8.790	9.427	8.439	15.811
lh_caudalanteriorcingulate_volume	0.103	4.83	−3.29	98.64	−14.70	−18.15	1.65	−19.30	0.756	0.605	0.449	0.080	0.333	0.252	0.355	12.045	2.835	10.770	3.534	14.325	8.463	14.201
lh_caudalmiddlefrontal_volume	0.273	77.30	13.55	−298.37	13.84	22.29	0.03	−119.28	**0.045**	0.383	0.348	0.495	0.625	0.994	**0.021**	2.390	4.983	10.438	8.908	14.065	1.977	1.264
lh_fusiform_volume	0.333	6.80	−25.60	85.92	19.77	64.05	4.20	−36.09	0.842	0.071	0.764	0.281	0.123	0.186	0.431	8.986	2.191	7.278	7.593	6.635	7.832	15.531
lh_inferiorparietal_volume	0.264	−28.99	−47.45	674.17	−18.66	66.68	9.30	91.99	0.663	0.084	0.227	0.599	0.404	0.133	0.302	13.914	2.352	9.527	7.956	15.429	5.835	12.684
lh_inferiortemporal_volume	0.384	70.59	−49.98	−113.59	−52.01	−38.86	7.48	−34.33	0.179	**0.022**	0.796	0.069	0.553	0.128	0.621	7.859	0.823	6.874	3.171	15.901	5.779	14.212
lh_lateraloccipital_volume	0.225	30.88	−24.99	−453.17	2.68	−1.34	3.13	−133.48	0.528	0.213	0.269	0.918	0.982	0.489	**0.044**	14.442	4.473	10.214	3.231	3.920	8.136	2.528
lh_lateralorbitofrontal_volume	0.418	−2.09	−11.53	−67.25	−20.50	11.12	2.71	−5.81	0.926	0.216	0.723	0.094	0.683	0.199	0.848	2.774	4.473	8.313	3.571	13.853	7.947	8.089
lh_lingual_volume	0.341	40.62	−53.23	−211.91	−48.23	−50.26	0.32	−73.16	0.234	**0.000**	0.456	**0.010**	0.220	0.919	0.110	9.589	**0.015**	10.770	0.530	10.348	4.547	5.854
lh_medialorbitofrontal_volume	0.420	2.20	0.73	−467.09	−13.57	−43.70	2.36	4.31	0.915	0.931	**0.009**	0.221	0.082	0.219	0.876	5.481	1.992	0.496	7.069	4.663	8.095	5.233
lh_middletemporal_volume	0.376	10.27	−53.61	−424.25	−26.94	−73.92	4.34	−51.70	0.817	**0.004**	0.255	0.258	0.169	0.291	0.385	9.746	0.197	10.094	7.725	8.430	8.479	14.884
lh_parahippocampal_volume	0.080	10.67	−3.39	−53.91	−2.49	1.32	2.15	−5.27	0.336	0.449	0.567	0.674	0.925	**0.038**	0.718	12.771	4.215	9.924	6.700	7.794	2.049	10.449
lh_precuneus_volume	0.225	15.32	−20.30	71.10	−15.18	−12.36	−0.73	−30.66	0.686	0.192	0.822	0.453	0.786	0.835	0.545	13.914	4.460	5.653	9.067	10.954	6.970	15.877
lh_superiorfrontal_volume	0.560	−16.84	−18.52	−393.05	−51.52	−49.45	4.87	40.91	0.768	0.406	0.397	0.091	0.461	0.341	0.594	11.345	4.983	10.314	3.568	16.152	8.490	15.437
lh_superiorparietal_volume	0.342	18.12	−13.63	626.43	−11.53	59.18	−3.44	−24.08	0.695	0.471	0.108	0.640	0.288	0.421	0.697	13.717	3.596	5.302	7.593	12.682	8.329	10.961
lh_superiortemporal_volume	0.377	80.50	−15.27	−892.17	−27.02	−51.48	4.36	−97.60	0.106	0.450	**0.034**	0.307	0.386	0.340	0.142	5.216	4.042	1.748	7.538	15.446	8.795	7.261
lh_supramarginal_volume	0.472	27.49	−55.79	141.18	−62.02	−85.06	−1.88	−29.43	0.471	**0.001**	0.657	**0.003**	0.067	0.593	0.564	15.081	**0.032**	8.679	0.193	3.999	8.297	15.283
lh_frontalpole_volume	0.046	−4.94	2.47	−26.05	−3.47	−1.43	0.08	−2.68	0.394	0.298	0.589	0.263	0.837	0.888	0.729	14.565	5.066	9.117	7.520	9.057	5.894	10.055
lh_temporalpole_volume	0.006	6.37	2.62	−92.36	−1.99	−10.54	2.09	−9.56	0.570	0.567	0.317	0.730	0.415	0.046	0.510	13.887	3.170	10.395	5.441	15.361	2.386	15.815
lh_insula_volume	0.451	−12.30	−0.81	−208.68	−20.12	−8.21	−0.04	33.76	0.620	0.936	0.315	0.132	0.782	0.986	0.310	14.041	1.863	10.486	4.736	10.954	3.675	12.703
rh_fusiform_volume	0.427	−29.07	−19.45	−408.09	−9.13	−9.65	−1.03	3.36	0.429	0.186	0.174	0.633	0.823	0.757	0.945	14.945	4.632	8.014	7.783	9.871	7.635	3.761
rh_inferiorparietal_volume	0.277	9.65	−66.57	−267.13	−14.18	31.49	5.34	−36.07	0.881	**0.014**	0.620	0.680	0.684	0.371	0.676	7.851	0.535	8.841	6.064	13.660	8.533	12.811
rh_inferiortemporal_volume	0.381	30.53	−19.13	−44.24	−24.16	−18.30	4.45	−9.74	0.489	0.303	0.904	0.302	0.728	0.283	0.868	15.164	5.066	4.265	7.549	12.146	8.707	6.888
rh_lateraloccipital_volume	0.260	7.94	−36.99	−100.51	−6.41	13.30	0.07	−26.80	0.872	0.071	0.808	0.808	0.823	0.988	0.685	8.425	2.191	6.370	4.038	10.214	2.959	11.544
rh_lateralorbitofrontal_volume	0.374	39.85	−12.16	2.86	−19.15	12.85	4.90	−25.26	0.146	0.275	0.990	0.189	0.694	0.054	0.487	6.852	4.956	2.895	6.440	12.999	2.760	15.594
rh_lingual_volume	0.250	57.60	−29.74	−361.29	−48.92	1.28	4.29	−33.78	0.154	0.073	0.283	**0.025**	0.979	0.249	0.529	7.102	2.128	10.395	1.336	5.789	8.463	15.877
rh_medialorbitofrontal_volume	0.454	−13.21	−2.94	−341.46	−11.35	−43.33	1.15	−1.66	0.427	0.664	**0.016**	0.203	0.033	0.453	0.940	14.945	2.420	0.906	6.688	2.004	8.163	4.379
rh_middletemporal_volume	0.393	−15.29	−52.10	−145.15	−40.84	−74.32	5.17	0.07	0.739	**0.005**	0.693	0.088	0.162	0.212	0.999	12.892	0.228	8.545	3.516	8.255	8.056	1.891
rh_parahippocampal_volume	0.258	6.86	−6.57	110.86	−3.18	17.50	1.62	−2.16	0.497	0.115	0.196	0.561	0.171	0.091	0.872	15.164	3.096	8.567	8.703	8.430	4.351	6.073
rh_paracentral_volume	0.288	13.75	−9.70	−99.02	−6.35	14.02	0.55	−24.44	0.510	0.257	0.570	0.568	0.576	0.774	0.382	14.905	4.883	9.647	8.412	15.540	7.573	14.884
rh_posteriorcingulate_volume	0.376	28.58	−14.37	−80.07	−5.89	25.99	2.98	−25.48	0.060	**0.022**	0.523	0.463	0.152	0.035	0.207	3.138	0.823	10.883	9.067	7.922	1.949	9.710
rh_precuneus_volume	0.332	−3.79	−24.52	269.03	−9.12	4.30	3.85	−26.42	0.925	0.138	0.423	0.670	0.929	0.300	0.622	3.678	3.579	10.565	7.042	7.398	8.365	13.671
rh_superiorfrontal_volume	0.423	−4.84	−29.03	−605.22	−39.49	−38.52	5.47	−29.53	0.947	0.331	0.321	0.311	0.660	0.416	0.762	1.853	4.628	10.144	7.357	13.853	8.329	8.761
rh_superiorparietal_volume	0.342	53.41	−39.94	397.73	−28.64	37.72	−6.31	−46.65	0.242	**0.034**	0.296	0.239	0.489	0.136	0.443	9.662	1.169	10.356	7.419	16.640	5.836	15.102
rh_superiortemporal_volume	0.401	86.53	−19.30	−404.29	−35.14	−28.70	8.52	−121.91	**0.021**	0.200	0.195	0.080	0.534	**0.015**	**0.015**	1.220	4.425	8.622	3.534	16.549	0.937	0.903
rh_supramarginal_volume	0.524	12.93	−33.93	182.60	−38.20	−27.98	8.11	−23.31	0.716	**0.022**	0.539	**0.048**	0.513	**0.016**	0.624	13.204	0.805	10.463	2.382	16.415	0.960	13.065
rh_frontalpole_volume	−0.024	0.91	2.92	−53.87	−3.42	−0.36	0.29	−6.64	0.894	0.306	0.354	0.354	0.965	0.659	0.470	7.047	4.856	10.138	7.795	6.501	8.573	15.510
rh_temporalpole_volume	−0.048	8.36	−4.76	−136.25	−1.84	11.66	−0.15	−6.54	0.555	0.412	0.252	0.808	0.494	0.909	0.730	14.438	4.867	10.094	4.519	16.640	5.326	9.475
rh_insula_volume	0.523	−41.52	5.97	−328.63	−27.90	−4.68	−0.84	66.88	0.077	0.528	0.093	**0.027**	0.866	0.694	**0.034**	3.825	3.296	4.639	1.394	8.367	7.973	1.963

*The Intra-cranial volume was considered in the model in order to normalize the brain volumes of all participants. Each variable was controlled for all other variables; R^2^, effect size (adjusted); β, beta; Values of p < 0.05 are considered significant and highlighted bold*.

## Discussion

Herein, we applied a multimodal whole-brain MRI approach to determine, in a normative aging cohort, the association between subjective sleep quality measured by PSQI global score, and brain connectivity. Results showed that poor sleep quality was associated with decreased FC and SC of two distinct networks, with overlapping nodes in the right superior temporal pole and in the left middle temporal region. The obtained data also showed that for older individuals, smaller increases in PSQI global score are sufficient to decrease FC in a network that has some overlapping nodes with the above-mentioned SC and FC networks. The left middle temporal region was significantly affected in all the found networks. Overall, the results indicate that network connectivity is adjustable in face of subjective sleep quality alterations and that a 1-month measure of sleep quality allows the observation of SC changes. In addition, the impacted networks, which are relevant for language, environment perception and assessment and self-awareness, seem to also permit inferences about a subjects' quality of sleep.

Across aging a concomitant increase in sleep complaints and individuals' vulnerability to psychological distress and medical conditions occurs (Bliwise, 1993; Bliwise et al., 1993; Maggi et al., 1998; Foley et al., 2004; Stranges et al., 2012; Scullin and Bliwise, 2015; Mander et al., 2017). However, prevalent as they are, it is still debatable whether these sleep complaints are a product of age itself, a consequence of age-related medical and psychiatric conditions, or a mix of both (Mander et al., 2017). Here, 49% of the participants had poor sleep quality over the previous month and of those, 34% were on benzodiazepines use and 28% used other type of psychopharmacological medication. In line with this result is the found association between PSQI subdomains “Day Dysfunction,” “Sleep Disturbance,” and “Medication” and depressive symptoms. In addition, participants with lower education levels had higher scores not only in PSQI subdomain “Medication,” which was also found in previous studies (e.g., Sivertsen et al., 2015), but also in GDS (depressive symptoms). In fact, more attention should be given to the weight of socio-demographic factors, given its association to health vulnerabilities, such as access to care or comprehension of health information. This is also relevant because it supports the concern about participants' cognitive and mental status and its influence in their ability to properly recall information. Herein, to address this limitation, PSQI information was combined with actigraphic measurement. A possible bias was addressed by the confirmation that no differences existed between the initial cohort and the subsample that used the ActiSleep+ units. The association between PSQI global score, its subdomains and actigraphic parameters was also tested. Important correlations between PSQI subdomain “sleep efficiency” and actigraphic TTB and TST were observed, as well as between sleepiness and WASO (actigraphy derived). No association was found between PSQI global score and actigraphy, which we speculate to be due to the combination of the small sample size and the number of variables tested. This limitation should be addressed in future studies by increasing the number of participants wearing actigraphy units. Additionally, because changes in body composition (e.g., increase of body fat mass and decrease of muscle mass) are common in aging (St-Onge, 2005), we also determined the distribution of BMI in our cohort and its association with sleep quality. Results showed that 56% of participants were overweight and 37.2% obese. These numbers are in line with the EuroStat information (Database–Eurostat^1^) that shows a high prevalence of overweight individuals from middle to older age. However, despite common, high BMI promotes an increased vulnerability to chronic diseases, such as sleep apnea, diabetes or cardiovascular diseases, which can further affect sleep quality (St-Onge, 2005; Hoevenaar-Blom et al., 2011).

In our study, we were not able to perform polysomnography to exclude non-diagnosed sleep conditions; we just relied on clinical information from their clinical processes. It is, therefore, important to state this as a limitation of the study. However, given that the purpose was to characterize a normative aging cohort, and it is common to have underdiagnosed sleep disturbances in the general population, we did not consider that this limitation would invalidate our study or its results.

The aging process also carries alterations to the neuronal system. In the present cohort, age-related changes in regional brain volumes occurred in areas involving cognitive function, motor behavior and emotional processing, which goes in line with other studies (Bernard and Seidler, 2014; Ritchie et al., 2015). Of note, we found a volumetric decrease of the putamen, which can be related not only to the aging process but also to the presence of depressive symptoms. Interestingly, the decrease of the volume of the left putamen was significantly associated with global cognitive decline in older individuals with memory complaints and Alzheimer's disease, exceeding the strength of the left hippocampal correlation to cognitive performance (de Jong et al., 2008). This is particularly relevant because our results show the association between the volume of left putamen and depressive symptoms and, in a previous work from our team (Santos et al., 2013) these depressive symptoms were a determinant factor for poor cognitive performance. Furthermore, despite no associations between regional brain volumes and PSQI global score were observed, some of the age-affected areas are relevant nodes in the FC and SC networks that in our study were found to be associated with subjective sleep quality. Thus, it is plausible to consider that age-related changes in brain regional volumes may be modulating not only SC, but also the FC of the networks associated to sleep quality. While it is easier to correlate brain volumetric changes with SC, the same does not apply to FC, given its plasticity, flexibility, and reorganization capabilities (Bullmore and Sporns, 2009; van den Heuvel et al., 2009; Tewarie et al., 2014; Meier et al., 2016; Fjell et al., 2017). We speculate that the age-related changes in brain regional volumes can modulate the number of connections between regions, which, in turn, will enable changes in regions that are working synchronously. We also cogitate that these can be a bidirectional relation, which means that it is possible that, at this point, we are also seeing the cumulative effect of disrupted (poor) sleep throughout lifetime and how much resilience people still have to it. Previous studies exploring the association between subjective sleep quality and brain parameters have used a single functional imaging modality. Here, we address the question with a multimodal approach, thus providing for a more comprehensive view of the phenomenon and casting some light in the still highly debated relationship between structural and functional brain connectivity. By complementing FC and SC we provide not only a measure of synchrony between regions (FC), but also evidence regarding the fiber connections and their integrity state (SC). Our results showed that three different networks were impacted by subjective sleep quality—two affected only by PSQI and the other by the interaction “PSQI × Age”—and that the left middle temporal region was the only node overlapping the three. When reflecting about the meaning of this result, two recent studies have to be considered: one from Van Someren and colleagues, in which results showed that medial temporal lobe atrophy was strongly associated to sleep-wake rhythm fragmentation (Van Someren et al., 2018); and another from Lauriola and colleagues, showing that despite the association between sleep disruptions and subjective cognitive decline, there was no correlation between sleep changes and the medial temporal lobe (Lauriola et al., 2017). Beyond the obvious methodological differences that can justify what seems to be contradictory results between these studies, another argument arises: maybe sleep changes precede medial temporal lobe atrophy (Liguori et al., 2017). In our results, the middle temporal region also presents a decrease of volume with age, so, maybe we are not only observing the effect of general age-related adjustments, but also age-related adjustments concerning chronic or cumulative effects of intermittent sleep disruption throughout lifetime. Furthermore, this region has been suggested to contribute to our ability to understand action and non-dominant semantic association, allowing semantic retrieval to be shaped to suit a task or context (Davey et al., 2016). Having this in mind, and despite that the real value of the finding is still uncertain, we hypothesize that the left middle temporal region, when connected to specific nodes of each of the found networks, may be shaping our sense of self and our sense of the world, by selectively retrieving information relevant for our action and context. In fact, these changes are observed in individuals with depression, schizophrenia or Alzheimer's disease, possibly supporting the bidirectional link between sleep and these pathologies. Furthermore, this could partially explain why improving sleep may help managing or even improving depressive, schizophrenic or Alzheimer's disease symptoms (Greicius et al., 2004; Onitsuka et al., 2004; Veer et al., 2010; Li et al., 2016, 2018; Yun et al., 2017; Shokri-Kojori et al., 2018; Son et al., 2018). Thus, the potential of these results for clinical practice is of relevance, as it opens new perspectives to intervene timely in face of sleep disturbances.

On the association between subjective sleep quality and FC, our results, indicate that poor sleep quality is correlated with a decrease in the synchronized activity of a network with important nodes the inferior parietal regions and left orbital middle frontal region. The FC of these areas was also found to be altered in other studies using sleep deprivation protocols or addressing good sleepers against poor sleepers or insomniacs (Sämann et al., 2010; Chen et al., 2014; Dai et al., 2014; Nie et al., 2015; Yeo et al., 2015; Krause et al., 2017), suggesting that these regions may have the potential to be markers of sleep disturbances. For example, it has been described that insomniac patients present an overall cortical hyperarousal during sleep, which is thought to reflect persistent sensory processing and subsequent shallower sleep (Desseilles et al., 2008). As a consequence, during wakefulness, these individuals present a decreased metabolism in subcortical (thalamus, hypothalamus and brainstem reticular formation) as well as in cortical regions (bilateral prefrontal cortex, left superior temporal, parietal, and occipital cortices; Desseilles et al., 2008). Our normative aged community-dwellers, in a restful wake MRI condition, also had a decreased connectivity in some of these regions, supporting the idea of a possible continuum from health to disease in sleep. A connection involving the inferior parietal region and the insula was also observed. The relevance of this finding is in the fact that the insula has a role in temporal and bodily states (Chen et al., 2014) integration, which in arousal networks may underlie the misperception of sleep quality and subjective distress in insomnia (Chen et al., 2014). In addition, the consistently impaired inferior parietal region has led to the hypothesis that this region may also be used as an early marker for the effects of 24-h sleep deprivation, serving as an indicator of unexplored behavioral impairments (De Havas et al., 2012). Some of the nodes of the networks associated to the PSQI global score are also relevant nodes for known networks, such as the default mode network, the attentional network and networks involved in reward, stress and social interaction. The structural connection patterns within the patterns of dynamic (“functional”) interactions (Sporns, 2003) showed that subjective sleep quality was also correlated to alterations in the SC of a network with nodes in the left lingual region, left caudate, left rectus and right medial orbitofrontal regions. These regions have also been previously described to be implicated in sleep disturbances (Kay et al., 2016; Kay and Buysse, 2017). Remarkably, in all the results that we had none seem to suggest that compensatory mechanisms exist in what concerns to poor sleep quality.

There are, however, some limitations that should be addressed, namely the ones imposed by the study design and sample size. While we have established novel associations between subjective sleep and brain properties, the cause-effect relation is still uncertain at this point. A longitudinal approach should be considered in further work. A larger sample size is also of need in order to incorporate other relevant variables that influence the sleep process, such as presence of relevant pathologies and use of medication and to promote a trait stratification analysis. The blood oxygenation level-dependent (BOLD) signal in functional MRI has been an increasingly used tool, but because it depends on hemodynamic parameters, like blood pressure, it is sensitive to medical conditions affecting the cardiovascular system. In our results, a weak but statistically significant association between diastolic blood pressure and PSQI global score was found. Since poor sleep quality is associated with increased vulnerability to cardiovascular diseases (Lao et al., 2018), we explored in our statistical model, whether diastolic blood pressure could be acting as a confounding effect. The results remained the same: the same networks were found and they were all statistically significant. On the other hand, the methodology employed in this study provides a novel perspective on the biology of sleep quality under normative conditions. Not only we considered a standard measure of sleep quality already in use in the clinical practice, but we also imaged whole-brain changes in MRI, thus allowing pinpointing the most relevant changes in the complex and dynamic brain networks. Moreover, by using a normative aging cohort, we managed to capture the associations of sleep quality with biological processes under daily-life conditions, which could provide a much more realistic view and understanding of sleep with all of its environmental interactions and thus, be of more utility in the design of possible future clinical interventions.

To sum up, the present study shows that middle-aged and older individuals in their normal aging process, display alterations in brain FC and SC of complex brain networks in association with subjective sleep complaints. This may be of relevance in the future, not only to design interventions that are effective in improving sleep quality but also to delineate studies that allow a better understanding of the mechanisms involving subjective sleep quality and its associated comorbidities.

## Author contributions

NS and NCS conceived the study and LA contributed to the study design. LA, TC, and CP-N performed participants' recruitment. LA and TC performed the psychological assessments. RM, PSM, and PM performed the MRI acquisitions and did the MRI data pre-processing. LA, RM, and AC perform the data analysis. LA wrote the first draft of the manuscript and all authors contributed for the following and final versions of the manuscript.

### Conflict of interest statement

The authors declare that the research was conducted in the absence of any commercial or financial relationships that could be construed as a potential conflict of interest.

## Footnotes

^1^Database–Eurostat. Available online at: http://ec.europa.eu/eurostat/web/health/health-status-determinants/data/database?p_p_id=NavTreeportletprod_WAR_NavTreeportletprod_INSTANCE_8D6M1FwWY36d&p_p_lifecycle=0&p_p_state=normal&p_p_mode=view&p_p_col_id=column-2&p_p_col_count=2 (Accessed January 6, 2018).
